# Carbolic Acid in Urine

**Published:** 1882-08

**Authors:** 


### SELECTIONS.

### CARBOLIC ACID IN URINE.

Dr. Tommassi (*L'Imparziale*, Dec. 1881.) gives the following method of testing for carbolic acid in urine, as being free from the fallacies of some other methods. First, prepare an acid solution containing 50

cub. centim. each of hydrochloric acid and of distilled water, to which add 20 centigrammes of potassium chlorate. Secondly, shake up equal volumes of the urine and ether in a test-tube ; let the ether rise, and decant it from the urine ; the ether contains now the carbolic acid. Thirdly, dip a chip of deal or bit of match-wood in the decanted ether till it is soaked ; then quickly into the acid solution ; then expose to sunlight. The presence of carbolic acid is shown by a blue coloration of the chip ; if there be none present, a faint greenish color may appear. One six thousandth part of carbolic acid may be thus detected. The chip must not be too long exposed to the sun, and the acid solution should be freshly made.—*Practitioner*, May, 1882.

